# High-throughput time-resolved morphology screening in bacteria reveals phenotypic responses to antibiotics

**DOI:** 10.1038/s42003-019-0480-9

**Published:** 2019-07-23

**Authors:** Taiyeb Zahir, Rafael Camacho, Raffaele Vitale, Cyril Ruckebusch, Johan Hofkens, Maarten Fauvart, Jan Michiels

**Affiliations:** 1 Centre of Microbial and Plant Genetics, KU Leuven—University of Leuven, Leuven, 3001 Belgium; 2 VIB-KU Leuven Center of Microbiology, Leuven, 3001 Belgium; 3 Department of Chemistry, KU Leuven—University of Leuven, Leuven, 3001 Belgium; 4 LASIR CNRS, Université de Lille, Lille, F-59000 France; 5 imec, Leuven, 3001 Belgium

**Keywords:** Microbiology, Imaging

## Abstract

Image-based high-throughput screening strategies for quantifying morphological phenotypes have proven widely successful. Here we describe a combined experimental and multivariate image analysis approach for systematic large-scale phenotyping of morphological dynamics in bacteria. Using off-the-shelf components and software, we established a workflow for high-throughput time-resolved microscopy. We then screened the single‐gene deletion collection of *Escherichia coli* for antibiotic-induced morphological changes. Using single-cell quantitative descriptors and supervised classification methods, we measured how different cell morphologies developed over time for all strains in response to the β-lactam antibiotic cefsulodin. 191 strains exhibit significant variations under antibiotic treatment. Phenotypic clustering provided insights into processes that alter the antibiotic response. Mutants with stable bulges show delayed lysis, contributing to antibiotic tolerance. Lipopolysaccharides play a crucial role in bulge stability. This study demonstrates how multiparametric phenotyping by high-throughput time-resolved imaging and computer-aided cell classification can be used for comprehensively studying dynamic morphological transitions in bacteria.

## Abstract (web summary)

In a high-throughput time-resolved microscopy screen, Taiyeb Zahir et al identify bacterial genes mediating morphological changes to antibiotics. An image analysis workflow enables the classification of single cells and deletion strains according to morphological changes.

## Introduction

Microscopy-based high-throughput screening for alterations in morphological phenotypes is a very powerful approach to systematically study biological processes and is one of the fastest growing techniques in cell biology^1,2^. High-throughput microscopy screening assays have been successful in finding genes involved in various biological processes^3–5^, building disease models^6^ and discovering proteomic changes induced by perturbations^7^. When the capabilities of this technique are augmented by live cell imaging at multiple time-points, it allows for a more in-depth analysis of dynamic processes^8,9^. Time-resolved imaging can be very useful to explore the dynamics of bacterial processes, such as morphological changes, induced by external perturbations. Filamentation and size minimization to evade host defense mechanisms^10^, spore formation upon starvation^11^, swarm cell differentiation^12^ are just few examples of shape transitions in bacteria due to environmental cues. Additionally, morphological changes in bacteria can also be induced by antibiotics and changes in gene expression^13,14^. Thus, the study of bacterial morphological changes in response to changing environments is invaluable for discovering novel gene functions, for chemical and genetic profiling of drugs or to explore heterogeneity and the role of cell shape in pathogenesis. High-throughput microscopy for studying the morphology of bacteria was recently described^15–17^, but these studies have been limited to single time-point imaging. Small feature size and rapid cell proliferation make it very challenging to perform high-throughput microscopy of bacteria in a time-resolved manner.

Here, we describe a combined experimental and image analysis approach for high-throughput measurement and analysis of morphological dynamics of large numbers of bacterial strains. Importantly, the high-throughput time-resolved imaging methodology is extremely timesaving and requires only off-the-shelf hardware and microscope software utilities. Furthermore, we establish a multivariate data analysis workflow for the classification of single cells into different morphological classes and for the characterization of the morphological dynamics of each strain based on the time evolution of morphological classes at the population level. We applied this methodology to assess the morphological changes induced by the β-lactam antibiotic cefsulodin in 4218 strains from the Keio collection^18^. The aforementioned workflow identified 191 genetic perturbations that produced significant phenotypic variation from wild-type *E. coli*. Functional analysis of these genes together with similarity-based clustering of their phenotypes revealed different types of atypical morphological dynamics and highlighted the cellular processes that can affect the outcome of cefsulodin treatment in *E. coli*. Our methodology opens the door for systematic large-scale studies of morphological changes in bacteria in response to changing environments. The simple experimental workflow and the comprehensive image classification approach described here is highly adaptable, providing an ideal platform for future high-throughput image-based studies of dynamic processes in bacteria as well as in diminutive cell types of higher organisms.

## Results

### High-throughput time-resolved microscopy imaging of bacteria

**Fig. 1:**
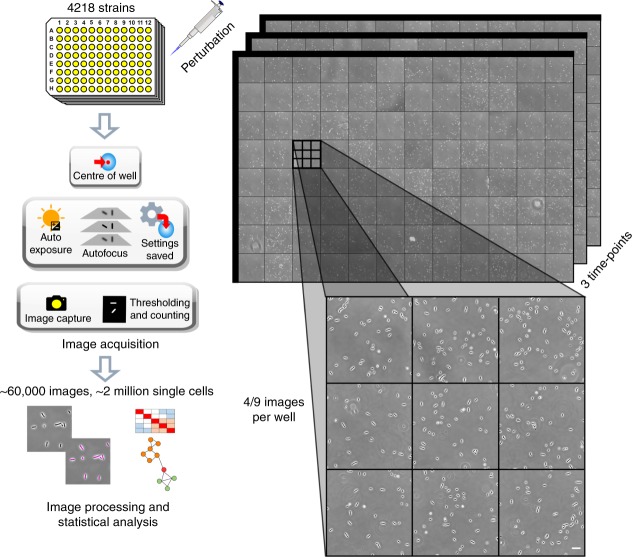
High-throughput time-resolved imaging methodology. Shown is a schematic representation of the high-throughput time-resolved imaging methodology. Strains are grown in glass-bottom 96-well plates and a perturbant is added to the desired wells. Morphological changes are monitored over time by automated phase contrast imaging. Image acquisition is dynamically regulated by automatically defining the exposure and number of images to be collected for every well depending on the cell density and cell morphology. Four or nine images were collected per well. The imaging protocol yields a rich dataset providing time-resolved records of phenotypes. In this work, 4218 strains from the Keio collection of non-essential single-gene deletion mutants of *E. coli* were perturbed by the antibiotic cefsulodin and images were recorded at three time-points leading to a dataset including approximately 60,000 frames and 2 million single cells. The image dataset is then subjected to image and data processing. Scale bar corresponds to 20 μm

Our goal was to develop a high-throughput methodology for the characterization and analysis of bacterial morphological responses to a perturbation of interest. To achieve this goal, we developed a high-throughput image acquisition workflow using off-the-shelf hardware and microscope software utilities (Fig. 1 and Methods). The workflow allows recording of bacterial morphological responses in the form of phase contrast images with single-cell resolution. Phase contrast microscopy is the most commonly used microscopy technique for capturing bacterial morphology. Phase contrast allows for the extraction of individual cell contours and monitoring morphological changes, such as cell lysis. We found that combining commercially available 96-square well glass-bottom plates with microscope air objectives (40X magnification; numerical aperture = 0.95; condenser phase stop Ph2, see Methods for details) allowed seamless multi-positional phase contrast imaging of bacterial cells suspended in liquid media for hours.

The sample preparation procedure is fairly straightforward and quick (Fig. 1). Strains are grown overnight in 96-well plates and diluted next day directly in the imaging microplate for re-growth. Strains growing in the imaging microplate are then subjected to a perturbation (for example, antibiotics) and the plate is kept on the microscope for imaging. The rigid and robust format of the microplates ensures that the spatial location of the strains remains constant during the experiment. Cells suspended in liquid media do not form a colony and stay separated, which facilitates accurate cell contour determination. Overall, the sample preparation requires minimal labor, comparable to any standard laboratory procedure done with 96-well plates.

We developed a completely automated image acquisition routine that is capable of obtaining optimal multi-position images from all 96 wells at multiple time-points without the requirement of any manual calibration or intervention (Fig. 1 and Methods). More specifically, it takes care of uneven focal position across the different wells and employs real-time analysis to optimize the image acquisition settings and the overall image acquisition workflow. Adaptation of the image acquisition settings is crucial to compensate for changes in cell density and morphology across wells and time. For all 96 wells, a set of operations are performed to find optimal image acquisition settings (Fig. 1 and Methods). Using these optimal settings, a single image is initially taken for each well to provide an estimate of cell density per well. The cell density is then used to determine the number of images that need to be taken at each well to reach a satisfactory number of cells for data analysis purposes. The time required to perform this task for the 96 wells is ~12 min. At this point, subsequent rounds of imaging can be performed at desired time intervals simply by utilizing the settings saved for each well. In summary, the imaging methodology described here is readily applicable to any large collection of bacterial strains and enables fast time-resolved imaging for quantifying dynamic phenotypes.

### Genome-wide screening of cefsulodin response in *E. coli*

**Fig. 2:**
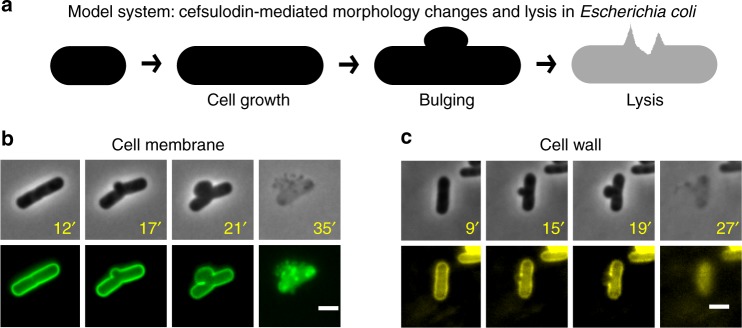
Morphological dynamics of *E. coli* in response to cefsulodin. **a** Schematic representation of the cell morphology changes induced by the β-lactam antibiotic cefsulodin. Cells lyse rapidly when exposed to cefsulodin. Lysis is typically preceded by bulge formation at the potential division site. **b** Cell membrane and **c** cell wall deformations in cells under the action of cefsulodin. Cell membranes are stained with the FM1-84 dye and the cell wall is visualized using the wheat germ agglutinin tetramethylrhodamine conjugate. Time stamps (in minutes) indicate the time after cells are seeded on cefsulodin containing agar pads. Scale bars correspond to 2 μm. The experiment was performed once

To demonstrate the capabilities of our method, we chose to investigate fast β-lactam antibiotic-mediated morphological changes in *E. coli* (Fig. 2a). Treatment of *E. coli* cells with β-lactam antibiotics results in rapid morphological changes and subsequent cell lysis^14,19,20^. Here, we use cefsulodin: a β-lactam antibiotic that inhibits cell wall building enzymes—PBP1A and PBP1B^21^. Cefsulodin-mediated killing of *E. coli* cells proceeds through two stages of morphological changes: elongation and bulge formation (Fig. 2a). Cells typically form a mid-cell bulge and lyse within 30–45 min of antibiotic exposure. The bulge formation is due to degradation and rupture of the peptidoglycan cell wall at the potential division site. As a consequence, the cell cannot keep its rod like shape and the inner and outer membranes of the cell are stretched outwards, forming the bulge (Fig. 2b, c). Figure 2b shows a cell undergoing lysis while labeled with the FM1-84 dye. FM dyes are known to label both inner and outer membrane in *E. coli*, albeit outer membrane much more strongly than the inner membrane^22^. Figure 2c shows the morphology of the cell wall (labeled with wheat germ agglutinin tetramethylrhodamine conjugate) at different stages of β-lactam-induced cell lysis. To find genetic factors that influence the morphological changes and lysis of *E. coli* to cefsulodin, we performed time-resolved imaging of the strains in the Keio non-essential gene knockout library^18^ (Fig. 1) after treating them with the antibiotic. Exponentially growing strains (around 5 × 10^6^ cells per ml) from the Keio collection were treated with cefsulodin in glass bottom 96-well plates and then imaged at three time-points corresponding to 30–38 min (T_30–38_), 47–55 min (T_47–55_) and 74–82 min (T_74–82_) after cefsulodin addition. The exact time depended on the location of the strain on the plate. The microscopy screening resulted in more than 60,000 images from three different time-points providing records of antibiotic phenotypic responses of 4218 strains.

### Image analysis and classification of cell types

**Fig. 3:**
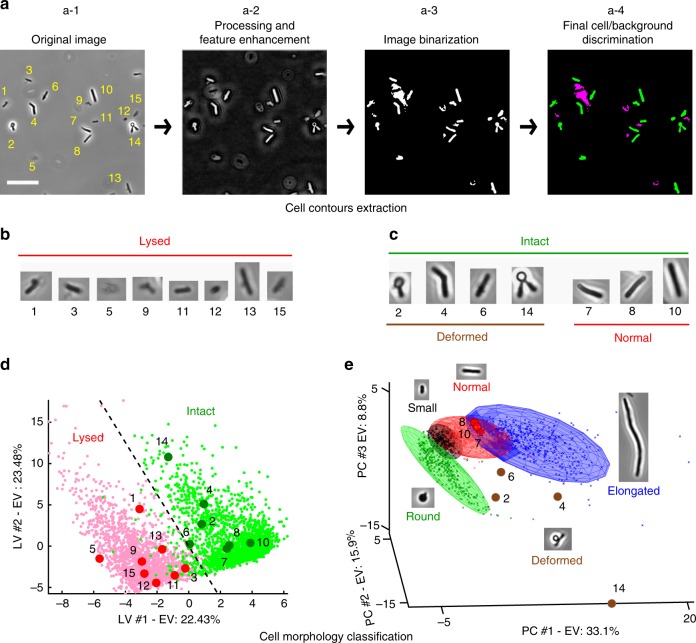
Classification of single-cell morphologies. Schematic representation of the image data analysis pipeline. **a** An example image is shown in a-1. Scale bar corresponds to 20 μm. Image segmentation consists of 3 main steps: processing and feature enhancement (a-2), image binarization (a-3) and image pruning (a-4, magenta). In the presented example, 15 single-cell contours are detected (a-4, green and numbered in a-1). **b**, **c** Images of all 15 cells obtained after segmentation. Eight cells were classified as lysed (**b**) and seven were classified as intact (**c**). Intact cells in **c** were further classified into two morphologies, i.e. deformed and normal. **d** Partial Least Squares Discriminant Analysis (PLS-DA) score plot of intact (large green dots) and lysed cells (large red dots). The model was built on a manually assigned set of 1728 lysed cells (small red dots) and 2017 intact cells (small green dots). The dotted line represents the direction of maximum separation between the two different classes of cells, perpendicular to the direction connecting the centroids of the two category point clouds. **e** Schematic representation of the results of the Soft Independent Modelling of Class Analogy (SIMCA) modelling, where the ellipsoids represent the 95% confidence interval of the round, small, normal, and elongated cell classes. Cells not assigned to any of these categories are classified as deformed. The SIMCA model was built on a manually assigned set of 332 round cells, 258 small cells, 595 normal cells and 376 elongated cells. In **d**, **e** cells from **b** and **c** are highlighted

In response to cefsulodin treatment, cells undergo morphological changes before lysing. To characterize the antibiotic response of each strain, we therefore computed the abundance and time evolution of lysed cells and different morphologies. To accomplish this, a multistep framework was developed to automatically classify cells based on their morphologies (Fig. 3 and Methods). First, images were processed to extract single-cell contours (Fig. 3a and Supplementary Fig. 1). For each cell contour, we computed various features/descriptors, such as cell length, cell width and aspect ratio (Supplementary Methods and Supplementary Fig. 2). Supervised classification was used to assess whether the cell had lysed due to antibiotic action and to determine the morphology of the cells that were intact (not lysed). First, intact cells were discriminated from lysed cells using Partial Least Squares Discriminant Analysis^23^ (PLS-DA) (Fig. 3b, c, d) and then the intact cells were classified into 4 morphological categories using Soft Independent Modelling of Class Analogy^24^ (SIMCA) (Fig. 3c, e). The morphological classes included cells showing normal, small, elongated and round morphology (Fig. 3e). Cells that did not belong to any of these four classes were assigned to the category ‘deformed’, which was found to encompass morphologies like elongated cells with bulges in the middle, constricted cells and fat or curvy cells. The procedure was applied on all images of the screening dataset. In all, 173 ± 81, 155 ± 69, and 164 ± 82 cell contours were analyzed per strain/well for time-point T_30–38_, T_47–55_, and T_74–82_, respectively. Minimum number of cells per strain/well was 50 (Supplementary Fig. 3). In total, around 2 million cells were automatically segmented and classified using this approach.

### Identification of genes affecting antibiotic response

To identify the mutants that show significantly different phenotypic responses compared to the wild type, we compared the dynamics of shape evolution in each strain with that of the wild type. The dynamics of shape evolution is represented by the proportion of the different morphological classes as a function of time. Therefore, we computed an 18-dimensional phenotypic profile for each strain (including the wild type). This profile consisted of the proportion of cells in 6 morphological categories i.e. intact, normal, round, elongated, small and deformed for each of the three time-points (columns G–X in Supplementary Data 1).

**Fig. 4:**
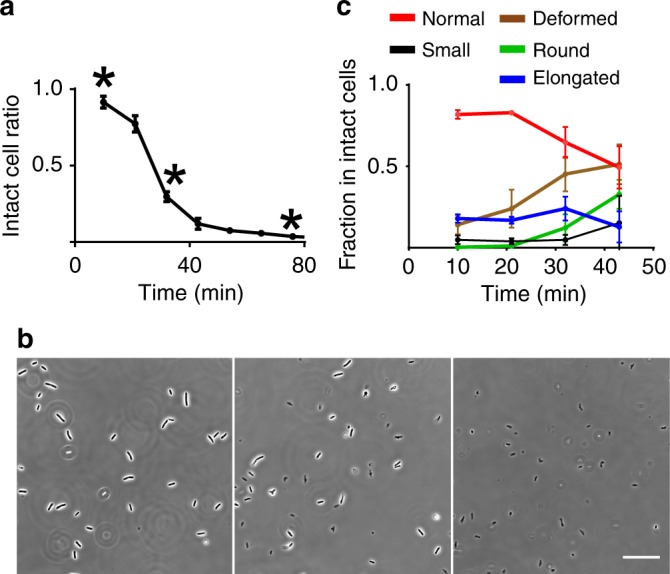
Response of wild-type *E. coli* to cefsulodin treatment. **a** Ratio of intact wild-type cells as a function of time after adding the antibiotic cefsulodin. **b** Images of the wild-type cells at three different time-points (marked by * in **a**). Scale bar corresponds to 35 μm **c** Fraction of cells belonging to each morphological class as a function of time. Error bars in **a**, **c** correspond to the standard deviation and the centers represent the mean estimated from *n* = 8 experiment replicates with 75–200 cells analyzed per experiment. Source data for panels a and b are available in Supplementary Tables 1–2

**Fig. 5:**
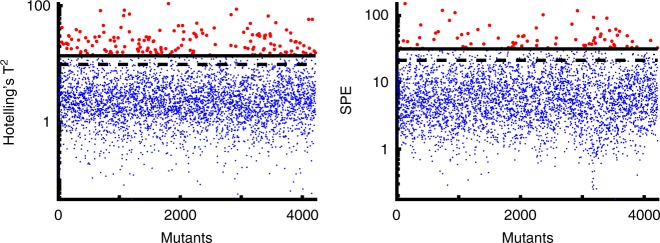
Quantitative analysis of phenotypic profiles of *E. coli* mutants treated with cefsulodin. Hotelling’s *T*^*2*^ and *SPE* values of all 4218 strains from the Keio collection from calculated from their phenotypic profiles. The dashed and the solid lines represent the 95th and 99th percentile, of the sample distribution of these two statistics observed for the wild-type control data (comprising 276 replicates), respectively. 191 abnormally behaving mutants that scored above the 99th percentile (shown as red dots) were considered for further investigation. Out of these 191 mutants, 32 showed only *SPE* values beyond the 99^th^ percentile of their respective reference distribution and 113 mutants showed only Hotelling’s *T*^*2*^ values beyond the 99th percentile of their respective reference distribution. The remaining 46 mutants showed values of both statistics beyond the 99th percentile of their respective reference distributions

Once the phenotypic profiles were obtained, we compared them against the wild-type response to identify mutants displaying atypical morphological dynamics. The wild type lyses in 45 min in presence of cefsulodin (Fig. 4a, b and Supplementary Table 1). As cells start to lyse, the proportion of deformed and round cells rises in the intact cell population, while the proportion of cells with normal morphology decreases (Fig. 4c and Supplementary Table 2). As a control, we determined phenotypic profiles for 276 wild-type replicates distributed over three microplates (or batches). Then, the phenotypic profile of each mutant strain and the wild type was assessed for dissimilarity based on two distinct statistics, Squared Prediction Error (*SPE*) and Hotelling’s *T*^2^ (Fig. 5 and Methods). A high *SPE* value indicates deviation from typical correlations between components of the phenotypic profile observed in the wild type. For example, if a mutant lyses slowly it displays a different correlation between the time-points and the intact cell proportion. High Hotelling’s *T*^2^ value indicates that the mutant displays a much higher or lower proportion of cells in a certain morphological category (or categories). One hundred and ninty-one mutants (Fig. 5 and Supplementary Data 2) that exhibited values of at least one of the two statistics beyond the 99th percentile of their respective sample distribution observed for the wild-type cells were considered for further investigation.

### Clustering of atypical phenotypic profiles

**Fig. 6:**
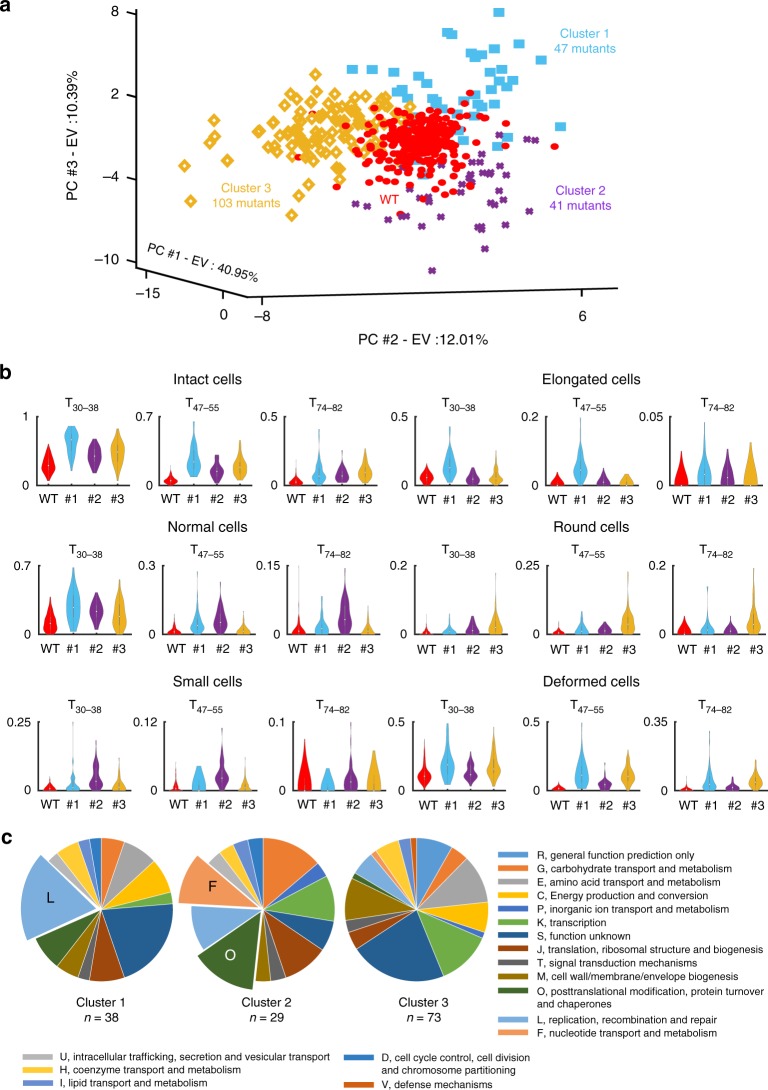
Phenotypic clustering of mutants displaying an atypical morphological response to cefsulodin. **a** PCA representation of the 191 mutants with atypical phenotypic profile, grouped using the k-means algorithm into three clusters. The number of clusters was determined by silhouette analysis. The wild-type phenotypic profiles are also shown as red dots for comparison. **b** Violin plots showing the distribution of the fraction of each cell type (on *Y*-axis) in the mutants belonging to the three clusters (#1, #2 and #3) at different time-points (T_30–38,_ T_47–55 and_ T_74–82_). WT refers to the 276 wild-type replicates used as a control. **c** Pie charts representing the relative distribution of Clusters of Orthologous Groups (COGs) in the three clusters of mutants. The enriched COG categories (*p*-value < 0.05; two-tailed Fisher’s exact test) are labeled and highlighted with a larger section of pie. The experiment was performed once for all mutants

To further characterize the phenotypic profiles of the 191 mutants that show an atypical response to cefsulodin, we performed a similarity-based clustering of the profiles using the k-means algorithm^25^. Cluster silhouette analysis^26^ revealed the presence of three major clusters of mutants (Supplementary Data 2). Figure 6a shows the principal component analysis (PCA) scores representation of the three clusters and highlights the different atypical phenotypic regions. Although these clusters resulted from the analysis of multiparametric profiles, they included phenotypes characterized by over-abundance (compared to the wild type) of certain types of morphologies. For example, several mutants in cluster 1 and 3 showed a high proportion of intact cells at the first two time-points (Fig. 6b). Cluster 1 mutants are characterized by the presence of normal, elongated and deformed cells whereas cluster 3 mutants by the presence of round and deformed cells. Cluster 2 mutants showed enrichment of small cells at T_30–38_ and T_47–55_ and normal cells at T_47–55_ and T_74–82_ (Fig. 6b). The enrichment of certain morphological types in these clusters highlights their different phenotypes. Over-abundance of normal and elongated cells points to impairment of processes that are required for cells to form a bulge. If mutants with cell sizes larger or comparable to the wild type proceed to bulging at a slower rate, they would show enrichment of elongated cells during the antibiotic action. On the other hand, mutants with cell sizes smaller than the wild type would form normally sized cells if there is an impairment in the process of bulging. This is illustrated by the mutants in cluster 2 that show enrichment of small cells in the beginning and normal cells towards the end of the lysis process (Fig. 6b). Enrichment of deformed and round cells indicates formation of stable bulges in the cells. When cells develop a bulge they are classified into the deformed morphology class. Enlargement of stable bulges gradually makes the cells appear as spherical, which classifies them into the round morphology class.

Clusters of Orthologous Groups (COG) assignment of genes deleted in the 191 mutants showed that each cluster encompassed genes belonging to various biological processes (Fig. 6c). Genes associated with categories N (motility), P (inorganic ion transport and metabolism) and Q (secondary metabolites biosynthesis, transport and catabolism) were absent or under-represented in the 191 mutants. Genes belonging to COG categories such as G (carbohydrate transport and metabolism), K (transcription), J (translation, ribosomal structure and biogenesis), and M (cell wall/membrane biogenesis) were present in all the clusters. For some categories, there were considerable differences between the clusters. For example, while category L (replication, recombination and repair) was represented in all the clusters, it was statistically enriched in cluster 1 (Fig. 6c). Similarly, cluster 2 showed enrichment of category O (Posttranslational modification, protein turnover and chaperones) and F (Nucleotide transport and metabolism). In cluster 3, several categories were either not present or under-represented including N, P, O, Q, and U (Intracellular trafficking, secretion and vesicular transport). Our analysis revealed distinct types of morphological changes during cefsulodin treatment. More importantly, the multiparametric data should enable discovery of novel associations and temporal relationships between different phenotypes. To test this hypothesis, we investigated the morphological dynamics that are associated with delayed lysis.

### Phenotypes associated with delayed lysis

**Fig. 7:**
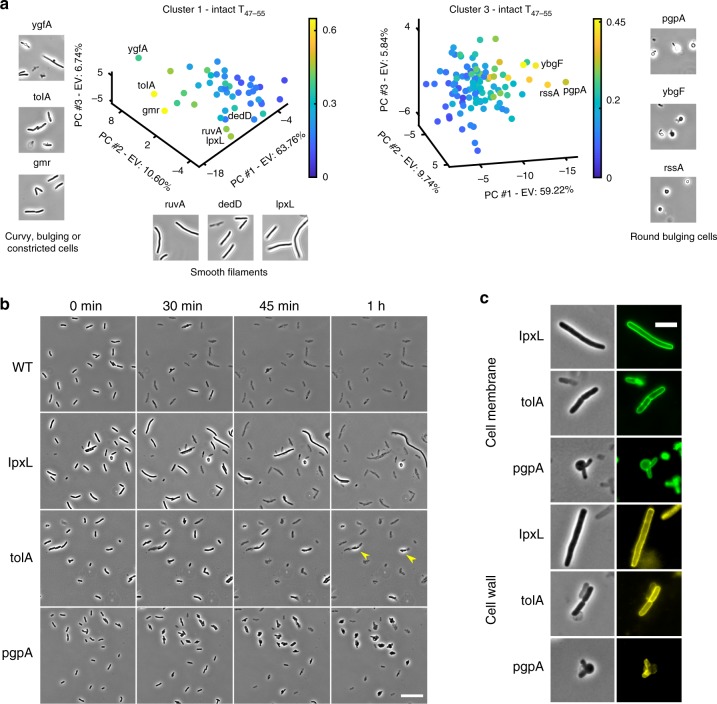
Phenotypes associated with delay in lysis of *E. coli* mutants following treatment with cefsulodin. **a** The plots show PCA representation of the phenotypic profiles of the mutants belonging to cluster 1 and 3. The color map represents the proportion of intact cells at time-point T_47–55_. Δ*ygfA*, Δ*tolA*, and Δ*gmr* display long bulging or constricted cells whereas Δ*lpxL*, Δ*dedD,* and Δ*ruvA* display long cells without constriction or bulge. Mutants with high intact cell ratio from cluster 3 (Δ*pgpA*, Δ*ybgF,* and Δ*rssA*) show cells with big bulges. **b** Micrographs show changes in morphology and lysis of the wild type, Δ*lpxL*, Δ*tolA*, and Δ*pgpA* cells on agar pads containing cefsulodin. Yellow arrowheads show an example of a cell growing in length and then lysing only half of its length. Scale bar corresponds to 20 μm **c** Cell wall and membrane morphology of Δ*lpxL*, Δ*tolA*, and Δ*pgpA* before lysis. Images were taken after 40 min of cefsulodin treatment. Scale bar corresponds to 5 μm

A high proportion of intact cells after antibiotic treatment implies delayed lysis. Clusters 1 and 3 (Fig. 6) contained all the mutants with a proportion of intact cells greater than 0.4 at T_47–55_ and greater than 0.2 at T_74–82_. Consequently, we probed the internal architecture of cluster 1 and 3 to find the responses of strains that showed delayed lysis (high proportion of intact cells at T_47–55_) and found 3 types of phenotypic responses in these mutants (Fig. 7a). Mutants that lysed late from cluster 1 were enriched in elongated and deformed cells and showed two major kinds of responses—overabundance of filamented cells with smooth periphery (for Δ*ruvA*, Δ*dedD* and Δ*lpxL*) and overabundance of long cells with bulges, curves and constrictions across their length (Δ*ygfA*, Δ*tolA* and Δ*gmr*) (Fig. 7a). On the other hand, mutants that lysed very slowly from cluster 3, such as Δ*pgpA*, Δ*ybgF,* and Δ*rssA,* formed large stable bulges and were thus enriched in cells with round morphology (Fig. 7a). We confirmed these different morphological phenotypes by performing time-lapse analyses on agar pads containing cefsulodin (Supplementary Figure 4). Figure 7b shows the response of the wild type and 3 mutants (Δ*lpxL*, Δ*tolA* and Δ*pgpA*) that displayed the most extreme case of the three atypical morphological dynamics. The wild-type cells lyse rapidly within 45 min of seeding on agar pads containing cefsulodin and rarely form stable bulges. In contrast, Δ*lpxL* cells elongate before lysing (Fig. 7b). Δ*tolA* cells also elongate, but with a constriction in the mid-cell position and lysis sometimes occurring on only one side of the cell constriction (Fig. 7b). Δ*pgpA* cells form very stable and large bulges (Fig. 7b).

These responses were further probed by determining the cell wall and membrane morphologies of cells after 30 min of adding cefsulodin. In case of Δ*lpxL*, we observed smooth cell wall and membrane morphology without septation and bulges after 30 min (Fig. 7c). This points to a delay in peptidoglycan degradation. Δ*tolA* cells showed fully formed septa composed of both the cell wall and the membrane. Δ*pgpA* cells displayed peptidoglycan degradation in the middle with no visible septa and the bulges were enclosed by the cell membrane. These results clearly demonstrate that our methodology is capable of identifying different atypical morphological dynamics and grouping strains/conditions exhibiting similar trends. Since β-lactam-induced killing culminates with cell lysis, the delayed lysis phenotype associated with distinct morphological changes could impede the killing process and confer antibiotic tolerance^27–29^. We chose to further investigate the mutants that showed the unusual stable bulging in the presence of cefsulodin to understand the implications of this phenotype on the antibiotic action.

### Stable bulging leads to antibiotic tolerance

**Fig. 8:**
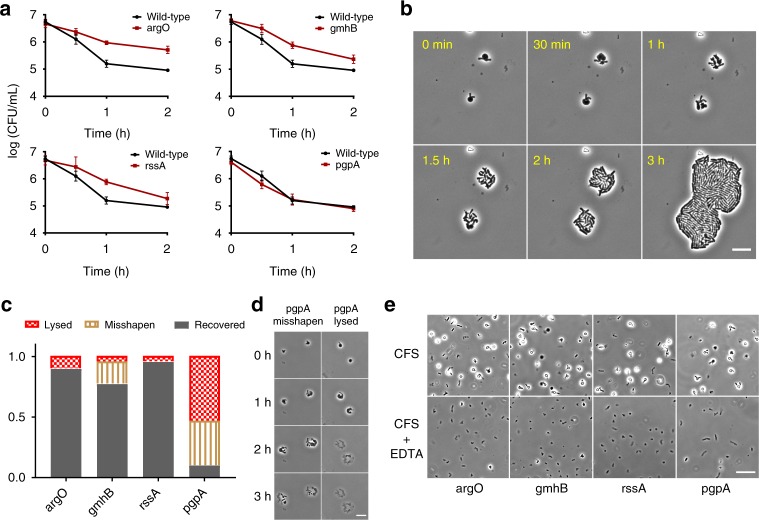
Cells with stable bulges can resume growth after antibiotic removal. **a** Time-kill analysis of the wild type and stable bulging mutants. Cefsulodin was added to a final concentration of 100 μg/ml at time 0. Error bars correspond to the mean ± standard deviation estimated from *n* = 3 experiment replicates. **b** Micrographs show the successful recovery of Δ*argO* bulging cells on antibiotic-free LB-agarose pads. Cells were collected after 1 h of exposure to cefsulodin. Scale bar corresponds to 10 μm. **c** Bars show the distribution of single-cell outcomes of regrowth in antibiotic-free medium for the stable bulging mutants. For each mutant, we recorded morphological changes in 30–45 cells. The outcome was determined manually from the colony morphology after 3 h of regrowth in antibiotic-free medium. Cells that lysed were put in the category of lysed, cells that could not revert back to rod-shaped cells were put in the category of misshapen and the cells that could form a colony of rod-shaped cells were put in category of recovered. **d** Micrographs show Δ*pgpA* bulging cells that could not successfully revert back to rod-shaped cells or underwent lysis on antibiotic-free LB-agarose pads. Scale bar corresponds to 10 μm. **e** Micrographs show the morphology of cells of stable bulging mutants after 1 h of cefsulodin (CFS) treatment with and without EDTA (1 mM) in the medium. Addition of EDTA in conjunction with cefsulodin abolishes stable bulge formation in the mutants. Scale bar corresponds to 20 μm. Micrographs in **b**, **d**, and **e** are representative of 1 experiment. Source data for **a**, **c** are available in Supplementary Tables 3–4

Stable bulge formation enables some strains to evade lysis by cefsulodin. A round cell morphology indicates stable bulge formation. We selected 15 mutants with the highest proportion of intact round cells at time-point T_74–82_ and compared their dynamics of round cell formation using the microplate imaging assay described above, but with more time-points. For 13 out of 15 mutants we found more than a five-fold enrichment of intact round cells compared to the wild type after 1 h of cefsulodin treatment (Supplementary Figure 5). Δ*argO*, Δ*gmhB*, Δ*rssA*, and Δ*pgpA* displayed stable bulge formation and the highest proportion of intact round cells after 1.5 h of treatment (Supplementary Fig. 5). We performed time-kill analyses on the wild type and the four stable bulging strains (Δ*argO*, Δ*gmhB*, Δ*rssA* and Δ*pgpA*) to assess the extent to which stable bulge formation can promote survival in the presence of cefsulodin. All the stable bulging strains except Δ*pgpA* showed higher survival rate than the wild type after 1 h of antibiotic treatment (Fig. 8a and Supplementary Table 3). The survival of cells with grossly altered morphology after antibiotic action not only depends on their ability to withstand lysis but also on their successful post-antibiotic recovery back to normally growing cells^30^. We suspected that post-antibiotic effects might explain the rapid killing of Δ*pgpA* despite the stable bulge formation, so we investigated the recovery of the stable bulging strains by collecting cells after 1 h of antibiotic treatment and seeding them on antibiotic-free LB-agarose pads. Time-lapse microscopy analysis showed that the bulging cells resulting from the antibiotic action were capable of producing normally growing cells after antibiotic removal (Fig. 8b). The majority of Δ*argO*, Δ*gmhB,* and Δ*rssA* cells grew in size and then successfully recovered to form colonies of rod-shaped cells (Fig. 8c and Supplementary Table 4). In contrast, the majority of Δ*pgpA* cells were defective in shape recovery and successful division after the antibiotic action (Fig. 8c and Supplementary Table 4). Cells that could not recover first grew in size and then lysed completely or underwent lysis accompanied by the formation of new cells that were not rod-shaped (Fig. 8d).

Previous studies have reported the use of divalent cations like Mg^2+^ for prevention of lysis^20,31,32^. The Mg^2+^ ions stabilize the outer membrane of bacteria by preventing the electrostatic repulsion between two adjacent lipopolysaccharide (LPS) molecules^33^. This prompted us to test whether disrupting the LPS layer affects lysis in the stable bulging mutants. Agents such as ethylenediaminetetraacetic acid (EDTA) can chelate the Mg^2+^ ions and severely affect the outer membrane integrity^34^. In presence of EDTA, the stable bulging mutants lost their phenotype (Fig. 8e). Together, these results show that cells with stable bulges can regrow back to normal cells when the antibiotic is removed and the integrity of the of the LPS layer is crucial in the formation of stable bulges during the antibiotic action.

## Discussion

In this work we established a workflow for high-throughput time-resolved imaging of bacterial strains and subsequent analysis of their morphological changes. Shape has profound effects on the bacterial life cycle and survival^35,36^. It is therefore not surprising that even though bacteria come in various defined shapes, they are capable of altering their morphology in response to environmental signals and as a part of their life cycle^37^. High-throughput measurement of morphological transitions in bacteria triggered by environmental cues has great potential for exploring how different morphologies convey a selective advantage in different environments and which cellular processes are crucial for adopting different shapes. We demonstrate that a rich set of information can be obtained by monitoring the morphological changes of cell populations in response to a perturbant. The proposed methodology ensures the coverage of many phenotypes and identifies genetic components that are important for a wide range of processes that are reflected in cell morphology.

We recorded morphological changes induced by cefsulodin in 4218 strains from the Keio collection^18^. Automated annotation of cell types converted the image data into phenotypic profiles containing information about the dynamics of the morphological changes in the population. Comparison of the phenotypic profiles with the wild type elucidated several gene deletions that altered the antibiotic response. Defects in DNA segregation and repair were associated with the enrichment of elongated cells. However, elongation of cells was likely not a response to cefsulodin, but simply a response to the gene deletions. The SOS response has previously been shown to delay cell lysis by β-lactams^38^. Defects in DNA segregation and repair can induce the SOS response and inhibit cell division^39^. Inhibition of cell division can delay the process of bulging and lysis^38^. This may explain the over-abundance of elongated cells in the SOS constitutive mutants such as Δ*xerD*, Δ*ruvA*, Δ*uvrD, ΔrecG, ΔdnaQ*, and Δ*rep*^40^. The *envC* and *dedD* deletion strains also show over-abundance of elongated cells. EnvC and DedD are involved in daughter cell segregation^41–43^. Deletion of these proteins causes a delay in splitting of the septal peptidoglycan between daughter cells. The enrichment of elongated cells in Δ*envC* and Δ*dedD* could thus be due to a delay in cell wall degradation. Several mutants have been described in the past to display filamentation or chaining phenotypes due to defects in daughter cell segregation. It is interesting to note that while gene deletions like *envC* and *dedD* that display smooth morphology when the daughter cells are attached^43,44^ were found to delay cell lysis, several of the chaining mutants that show an indentation between attached daughter cells including Δ*tatA*, Δ*tatB*, Δ*tatC*, Δ*pdxH*, and Δ*amiC*^17,45,46^ displayed rapid cell lysis to cefsulodin. This indicates that the cefsulodin-mediated lysis begins at the onset of peptidoglycan degradation during cell segregation.

We also found some striking results such as the late lysis phenotype of *tolR*, *tolA*, and *ybgF* deletion strains. TolR, TolA and YbgF are part of the Tol-Pal complex in *E. coli* and deletion of any of the components compromises the cell envelope integrity^47^. As such, it is counterintuitive that deletion of these genes would provide cell envelope stabilization after β-lactam treatment. Deletion of *tol-pal* genes also causes late invagination of the outer membrane during cell division, possibly due to a delay in cell wall degradation^48^. This could explain the phenotype of late lysis. Another striking result is the filamentation phenotype of strains such as Δ*lpxL* and Δ*dedD*. It is widely accepted that inhibition of PBP1A and 1B by cefsulodin cause rapid lysis in *E. coli* and inhibition of PBP3 by antibiotics, such as cephalexin and aztreonam, causes filamentation^49^. While this is generally the case for most of the mutants, we observed that deletion of genes such as *lpxL* and *dedD* causes *E. coli* to filament as a response to inhibition of PBP1A and 1B.

In this work, we discovered several single-gene deletion strains that can form giant stable bulges upon treatment with cefsulodin. Their phenotype is reminiscent of how spheroplasts are formed when bacterial cells are kept in osmoprotective conditions and their cell wall synthesis is inhibited^20,50^. These mutants can evade cefsulodin-mediated lysis without the requirement of osmoprotective conditions, and the LPS layer of the outer membrane plays a crucial role in the formation of stable bulges and escaping lysis. Furthermore, we show that cells with stable bulges can revert back to normally growing cells after transient exposure to cefsulodin. Thus, stable bulge formation can confer antibiotic tolerance to these strains.

The high-throughput microscopy technique we described here is designed to facilitate fast time-resolved imaging. While we imaged cells suspended in liquid media, previous high-throughput microscopy studies used large format agar pads as the substrate to seed bacteria for live cell imaging^15,16,51^. In general, preparation of large agar pads requires time and labor, and they are prone to drying and shifting during long time-lapses. Drying of agar pads changes the spatial position of strains and the problem can be severe for large agar pads. Although imaging on agar pads provides more accurate cell contours, there are some limitations of using agar pads for time-resolved imaging. These limitations mostly originate from cell growth over time. Cell contours extraction becomes tricky on agar pads as cells grow to form a colony. Also, bacterial physiology changes considerably after the end of steady-state growth (optical density at 595 nm ≈0.3 for *E. coli*)^52^ and unfortunately the preferred cell density for high-throughput imaging on agar pads is often very close to the cessation of the steady-state growth phase^15^. Cessation of steady-state growth can cause changes in morphology that are non-specific to the perturbant. For example, β-lactam mediated cell lysis kinetics depends on the growth rate^53,54^ and the growth rate changes abruptly after the end of steady-state growth phase. Therefore, it is imperative to work with low cell densities so that the growth rate stays steady throughout the whole process of β-lactam-induced lysis. Our high-throughput imaging setup enables collection of single-cell measurements for robust data analysis at cell densities much below the cessation of the steady-state growth. As such, this setup is very suitable for selective measurement of morphological changes that are direct consequences of the perturbant and not the growth phase of bacteria. The imaging methodology is meant for monitoring bacterial strains for long time-periods. The protocol can be adjusted in a variety of ways to allow imaging for a certain duration of time. For example, by decreasing the initial cell density and capturing more images per well, the duration of imaging can be increased while still obtaining sufficient measurements for data analysis purposes. We observed that a cell density of more than 125 normal shaped *E. coli* cells in the field-of-view caused aggregation of cells and problems with cell contour extraction. Even for rapidly multiplying bacteria (generation time of 20 min) and a requirement of minimum 30 cells at every time-point, capturing 9 images per well allows imaging and subsequent analysis for around 2 h.

We believe that the image and data analysis framework that we described here can be implemented to any bacterial image dataset by training the classifier on specific subsets of cellular features. Cells suspended in liquid media are motile. This does not affect the analysis, because we compute phenotypes from the frequency of morphologies in the cell population. But, it precludes microscopic measurement of changes in single-cell physiology over time (like in a time-lapse). Our rigorous analysis ensured very accurate automated phenotyping of single cells. We have used some novel features to classify single cells in this work, such as Fourier descriptors (a relatively new class of shape descriptors) which are invariant to translation, rotation and scaling^55^ and microenvironment features that were calculated by taking into account both the intensity of the cell and the intensity of its immediate surrounding. Through multiparametric analysis we were able to identify several gene deletions that yielded atypical responses to cefsulodin. Furthermore, we also propose ways for downstream analysis. Similarity-based clustering of hit genes elucidated three major types of atypical responses and found enrichment of genes belonging to particular cell processes within a cluster. The internal architecture of each phenotypic cluster revealed information regarding phenotypes associated with enhanced cell survival. Our morphological classification approach revealed subtle phenotypic distinctions. For example, we could discriminate between the mutants enriched with smooth long filamented cells and the mutants enriched in constricted or bulging long cells even though both phenotypes caused cells to grow longer. Achieving this degree of distinction highlights the large amount of information that can be derived from high-throughput microscopy image datasets when appropriate analysis techniques are implemented.

In conclusion, to the best of our knowledge we report the first image-based systematic study of dynamic morphological changes in bacteria for a large collection of strains. Our method can be complemented by several genome-scale bacterial libraries^18,56–58^ for high-throughput studies that are similar to forward genetic screenings. The approach is generally applicable for characterizing single-cell morphological changes due to a plethora of external stimuli and internal cellular states. This work provides a platform for future studies, including the possibility of incorporating dyes and fluorescent proteins to reveal subcellular protein localization, gene expression changes, structural changes in cellular components etc., in bacteria as well as in diminutive cell types of higher organisms.

## Methods

### Bacterial strains and growth conditions

High-throughput microscopy screening was performed using the ordered Keio collection of non-essential single-gene deletion mutants of *E. coli*^18^ and all the strains investigated further also came from the same library. All experiments were carried out at 37 °C in Luria-Bertani (LB) medium.

### High-throughput microscopy screening

96-well plates with glass bottom (Brooks Automation Ltd. MGB096–1–2-LG-L) were used for high-throughput imaging. When bacteria are close to a glass surface below them, they tend to stay near the surface^59^. In case of glass-bottom 96-well plates, this leads to accumulation of cells near the bottom of wells. The use of microplates, thus enables high-throughput imaging even at low cell densities because the majority of cells in the liquid culture settle down at the bottom of the well and by adjusting the culture volume in the well, one can get satisfactory numbers of cells at the imaging plane even for low cell densities. Imaging at low cell densities (optical density at 595 nm < 0.3) is important to avoid changes in growth rate and morphology over the time course of imaging due to factors other than the perturbation itself, like cessation of steady-state growth^52^. Imaging microplates are available in 96 wells, 384 wells and even higher density formats. 96-well plates with wide square wells are compatible with phase contrast microscopy. Plates with a higher density of wells do not allow the light cone from the phase annulus to pass through. The Nikon software’s JOBS plugin was used to construct an image acquisition routine that controls automated movements of the microscope hardware. The routine also performs all the necessary steps required for obtaining the well-specific settings. First, the auto exposure is executed to correct for brightness differences due to varying numbers of cells in different wells. For auto-exposure on each well, the over-illumination tolerance is set to 30%. To correct for variations in glass bottom thickness, the Z-offset is determined for each well. To determine the Z-offset, a 6 μm deep Z-stack with a step size of 1.2 µm is collected above and below the plane of interest defined by the perfect focus system. The offset is selected based on maximum contrast between cells and background. Using the Z-offset and exposure settings, one image per well is collected for defining the desired number of images to be recorded in subsequent rounds of imaging. These well settings (exposure, Z-offset, number of images) are then saved for each well. Stage movement is kept in the meandering mode with respect to the lens (horizontal) and multiple imaging positions inside the well are kept in a grid to reduce the imaging time for the whole plate. With this approach, it takes ~7 min to capture four images from all 96 wells. The robust structure of microplates ensures that the well Z-offset remains constant throughout the time-course of experiment. For subsequent rounds of imaging, optimal exposure calculation is incorporated to compensate for changes in cell count and morphology.

### Screening for morphological responses to cefsulodin

The screening was performed with a Nikon Ti-E inverted microscope equipped with a Qi2 CMOS camera and a temperature-controlled cage incubator. Images were acquired using an air objective (CFI Plan Apochromat Lambda DM 40X; NA = 0.95; Ph2). All the strains from the Keio library were grown overnight in 96-well plates (Greiner, 96-Well Polystyrene Microplate, clear) containing LB with 30 µg/ml Kanamycin (MP Biomedicals), and then diluted 5000 times in 96-well plates with glass bottom (Brooks Automation Ltd. MGB096-1-2-LG-L) containing 100 µl of fresh media. Cells were grown for 2 h in standard incubators with continuous shaking and then treated with 100 µg/ml (final concentration) of cefsulodin (Sigma-Aldrich). The plates were then covered with a plastic lid and kept on the microscope stage for imaging. Plates were not shaken during or between the microscopy measurements. Since most cells settle down at the bottom of the wells, the focal plane for imaging was just above the glass bottom. During the entire course of the experiment, the perfect focus system was kept in the ON state. A 5 min waiting period was inserted before starting the imaging procedure to allow cells to settle at the bottom of the wells. First, optimal image acquisition settings were calculated for each well and one image was recorded simultaneously using the optimal settings. We did not include this image in the data analysis, however the number of cells segmented in this image was used to decide the number of images required from each well. Nine images were taken from the well if there were less than 15 cells, otherwise four images were taken.

### Image processing

Segmentation of bacterial cells from the images was challenging due to the substantial local differences in intensity and also because the antibiotic action resulted in cells with different morphologies and intensity profiles. First, the contrast between cells and background was enhanced by adaptive histogram equalization and top-hat filtering. Then, an appropriate cutoff value was calculated to binarize the image. Since we wanted to segment both intact cells (with high contrast to background) and lysed cells (with low contrast to background), this step was not very stringent. As a consequence, some non-cell features were also present in the binarized regions. Therefore, in a final step we pruned regions that did not pass pre-defined minimal cell shape criteria. For every cell, 54 features were computed that quantified their intensity, shape and microenvironment characteristics (see Supplementary Information). The cell descriptors served as input for the supervised classification of cells based on their morphology.

#### Data analysis

Cells were first classified as lysed or intact using a well-established discriminant technique, namely PLS-DA^23^. The PLS-DA model for intact vs lysed cell discrimination was calibrated on a training set containing the values of the 54 descriptors measured for 3745 cells, randomly selected from various images captured at different time points during treatment with cefsulodin. Validation performed by exploiting an external representative test set of 1650 cells yielded satisfactory results (Supplementary Table 5) in terms of both classification sensitivity (true positive rate) and specificity (true negative rate). Intact cells were further classified into 4 morphological classes—small, normal, round and elongated. Given the extreme heterogeneity in the morphology of bulging cells, we included all cells that did not belong to any of the four target classes into the category of deformed. We used SIMCA^24^ to categorize cells based on their shape. The training set included 376 elongated cells, 595 normal cells, 332 round cells, and 258 small cells. Cell classification accuracy based on the cellular descriptors, was estimated by seven-fold cross‐validation to be 82–99%, depending on the cell class (Supplementary Table 6).

Both the PLS-DA and SIMCA models were trained using wild-type cells. Residuals analysis highlighted that both models were reasonably extendable for the determination of the intact/lysed status and morphological category of mutant cells. This is understandable because cells with similar morphologies are supposed to yield similar values of the intensity and shape descriptors even when they have differences in their genome. The proportion of cells in every morphology class was determined based on the abundance in the whole population including both intact and lysed cells. Prior to data analysis, we corrected the data from all plates containing the wild type and the mutants for batch effects (Supplementary Fig. 6). The correction is performed by centering and scaling to unit standard deviation data from each plate separately.

The 18-dimensional phenotypic profile consisted of the proportion of cells in six categories (intact, small, normal, round, elongated and deformed) at three different time-points. The 276 phenotypic profiles of the wild type were subjected to PCA in order to build a data-driven mode,l which captured the main variability associated to the in-control behavior of wild-type cells. The main idea here is to utilize this model for spotting clearly outlying behaviors among the mutants. The degree of outlyingness of each mutant phenotypic profile was estimated based on two distinct statistics, Squared Prediction Error (SPE) and Hotelling’s *T*^2^. *SPE* reflects the perpendicular (orthogonal) distance between its respective observation vector from the PCA model hyperplane, while Hotelling’s *T*^2^ is the Mahalanobis distance between the origin of the PCA model hyperplane and the projection of its respective observation vector onto it. In practice, abnormal values of *SPE* would identify breakages in the correlation structure underlying the different descriptors under study as observed in the wild-type experiments. On the other hand, abnormal values of Hotelling’s *T*^2^ would highlight wells exhibiting extreme values in some (or all) of the considered parameters, but preserving their original correlation structure. Confidence limits for *Q* and Hotelling’s *T*^2^ can be calculated according to Qin et al.^60^. Here, all the mutants found to exhibit values of at least one of the two statistics beyond the 99th percentile of their respective sample distribution observed for the wild-type cells were considered as outliers.

### Time-lapse and fluorescence imaging

Time-lapses of the wild type and mutants were performed on LB-agarose (2%) pads containing cefsulodin. Strains were grown overnight, then diluted 1:1000 next day in glass test tubes containing fresh LB and grown for 2 h. Cells were then seeded on the agarose pads for imaging. For cell wall and membrane morphology assays strains were grown overnight, diluted 1:1000 in fresh LB, grown for 2 h and then treated with cefsulodin in glass test tubes. After 30 min of cefsulodin treatment, appropriate amounts of dyes were added to stain the cell envelope. Cell wall was labelled using 25 µg/ml of Wheat Germ Agglutinin, Tetramethylrhodamine conjugate (Thermo Fisher). For labelling the membranes, 2.5 µg/ml FM1-84 dye (Biotium) was used. After staining, cells were seeded on LB-agarose pads for imaging. To show post-antibiotic recovery/non-recovery of bulging cells on agar pad, cell cultures were grown overnight. The next day, cells were diluted 1:2000 in fresh LB and grown for two additional hours. Cefsulodin was added to a final concentration of 100 μg/ml and the cell culture was kept at 37 °C non-shaking environment for an additional 1 h. After the antibiotic action, the cells were diluted 1:20 in antibiotic-free media for 5 min to lower the antibiotic concentration. Cells were then seeded on LB-agarose pads for microscopy. For observing the lysis behavior of stable bulging mutants in presence of EDTA, cell cultures were grown overnight, diluted 1:5000 in two wells containing fresh LB and grown for additional 2 h. After this, 1 replicate well was treated with only cefsulodin and another replicate was treated with cefsulodin and EDTA (1 mM) simultaneously. Images of cells were obtained after 1 h of treatment. Cell envelope morphology experiments were done on a Zeiss Axio Imager.Z1 fluorescence microscope. All the other images were taken on a Nikon Ti-E inverted microscope.

### Antibiotic time-kill measurements

For time-kill experiments, strains were grown overnight and then diluted 1:2000 in fresh media to grow for an additional 2 h. Then cefsulodin was added to a final concentration of 100 μg/ml. Cultures were afterwards incubated in a 37 °C static environment. Aliquots of 1 ml were taken at appropriate time-points and washed in 25 mM MgSO_4_ solution. Cell suspensions were then serially diluted and plated on LB agar plates. Cell survival was measured by CFU (Colony Forming Units) counts. All the experiments were repeated thrice.

### Cluster of orthologous groups analysis

COG categories were assigned to all the mutants using the eggNOG tool^61^. The enrichment analysis was carried out by two-tailed Fisher’s exact test and all COG terms that showed a significant enrichment (*p-*value < 0.05) are shown in Fig. 6.

### Turbidimetric studies

Optical density measurements were collected using a Synergy Mx Multi-mode Microplate Reader (Biotek). Overnight grown cultures were diluted 1:1000 in LB and further grown in Greiner 96-well plates for 2 h. Then, cefsulodin was added to a final concentration of 100 µg/ml in the wells and the OD was monitored at regular time intervals. OD was measured at 595 nm.

### Statistics and reproducibility

Image data was first analyzed to find the number of cells imaged for every mutant. Mutants/wells with less than 50 cells in total or more than 150 cells per frame were repeated. Data from every plate was preprocessed to correct for batch effects. Every well/mutant data was normalized using the data from the whole plate. Three components are used for the PLS-DA model. For SIMCA analysis, the number of components used are: one for elongated cells, one for normal cells, three for round cells and two for small cells. The number of components in all cases were assessed by seven-fold cross validation. We achieved a high specificity and sensitivity (more than 81% for every cell class). Data analysis was performed without prior knowledge of the arrangement and order of strains. Many extreme phenotypes were validated either by microscopy on agar pads or using the 96-well plate assay but with more time-points.

The CFU counting experiments were performed three times. Results are expressed as a mean ± SD. COG enrichment was tested by two-tailed Fisher’s exact test. *p* values < 0.05 were considered to be statistically significant.

### Reporting summary

Further information on research design is available in the Nature Research Reporting Summary linked to this article.

## Supplementary information

Description of Additional Supplementary Files

Supplementary Information

Supplementary Data 1

Supplementary Data 2

Supplementary Code 1

Reporting Summary

## Data Availability

The authors declare that the data supporting the findings of this study are available within the paper (and its Supplementary Information files).

## Code availability

Code availability The computer codes are available within the paper as supplementary files called “Supplementary Code 1”.
