# Supplementary material for: High-throughput time-resolved morphology screening in bacteria reveals phenotypic responses to antibiotics
Source: Commun Biol. 2019 Jul 23;2:269. doi: 10.1038/s42003-019-0480-9 (PMC6650389; doi:10.1038/s42003-019-0480-9)
Supplement: Supplementary file 1 — Description of Additional Supplementary Files [file 42003_2019_480_MOESM1_ESM.docx]

**Description of additional supplementary items**

**File Name:** Supplementary Data 1

**Description:** The table contains the results from the microscopy screen. For every mutant the ratio of different cell morphotypes at different time-points are given. In addition, the SPE and *Hotelling’s T^2^* values are also listed.

**File Name:** Supplementary Data 2

**Description:** The table contains all mutants with SPE and/or *Hotelling’s T^2^* scores above the 99^th^ percentile of the wild type. The results of k-means clustering is also shown in a separate column. 3 clusters were identified and the column C indicates the cluster to which the mutant belongs.

**File Name:** Supplementary Code 1

**Description:** The zip file contains all the codes used for image and data analysis. main_01_Cell_Seg_several_plates.m, main_02_SDCalculation_several_plates.m, main_03_LDclassification_several_plates.m, main_04_ShapesClass_several_plates.m and main_06_ShapeCmp.m should be run sequentially on the folder containing the entire image data set.

main_01_Cell_Seg_several_plates.m segments single cell contours

main_02_SDCalculation_several_plates.m calculates the single cell descriptors

main_03_LDclassification_several_plates.m classifies all cells into intact and lysed

main_04_ShapesClass_several_plates.m performs shape classification of the intact cells

main_06_ShapeCmp.m performs SPE and *Hotelling’s T^2^* calculations on every mutant
