# Supplementary material for: High-throughput time-resolved morphology screening in bacteria reveals phenotypic responses to antibiotics
Source: Commun Biol. 2019 Jul 23;2:269. doi: 10.1038/s42003-019-0480-9 (PMC6650389; doi:10.1038/s42003-019-0480-9)
Supplement: Supplementary file 2 — Supplementary Information [file 42003_2019_480_MOESM2_ESM.pdf]

Supplementary Methods

The successful segmentation of bacterial cells in the images is a step of paramount importance for the proposed methodology. In this step the cell contour, later used for the calculation of shape descriptors, is defined. In the case of our experiments, with *E. coli* in suspension the phase contrast images are characterized by:

i. A relatively low density of cells (akin to low confluency);
ii. Intra- and inter-frame intensity variations. These variations arise from local sample variations and the use of auto-exposure during image acquisition. Note that while auto-exposure induces inter-frame intensity variations, it also maximizes the contrast of each frame;
iii. Substantial changes in morphology of cells;
iv. Intensity variations depending on the status of the imaged cells. An intact cell is characterized by a darker-than-background body surrounded by a brighter-than-background halo. On the other hand, a lysed cell is characterized primarily by a darker-than-background body with a significantly lower contrast than an alive cell.

Segmentation was carried out by first implementing a contrast-limited adaptive histogram equalization that locally enhances contrast while keeping the overall image homogeneous. Then, in order to highlight the darker-than-background features of the original image, its complement was calculated. Finally, top-hat filtering was used to further increase image quality, particularly for removing unwanted background fluctuations.

To find the appropriate binarization threshold value (signal/background) for each image we developed a procedure akin to Otsu's method. Under the assumption of low cell density, we expect

that a large part of the image contains background pixels. Furthermore, as a consequence of the image enhancement procedure, cells are brighter than background. Therefore, we generated a histogram of the pixel values, and performed a Gaussian fit to the low intensity region of the resulting distribution (i.e. to the background intensity values). The image segmentation threshold was then calculated according to the Full-Width-at-Half-Maximum (FWHM) obtained for this background intensity distribution.

Once binarization is completed, extra processing steps are executed to improve the quality of the final output. Segmented regions that are in contact with the image border are removed, as their morphology cannot be accurately defined. Additional imperfections and artifacts are filtered out via image opening with a disk structural element.

As a final step, some basic morphological properties of the segmented regions are checked to remove those that do not comply with specific expected parameters, for example, width, aspect ratio, intensity, presence/absence of a halo.

Definition of the single cell features (or descriptors) used in this study

Intensity-based descriptors (total number: 33):

An important feature of *E. coli* cells when imaged via phase contrast microscopy is that their body is darker than the background. Furthermore, in the images, the intact cells are surrounded by an intense halo which results from the diffracted light passing through the phase ring as well as the large difference in the optical path between light going through the intact cells and the immediate surrounding background. This means that the pixels located outside the cell boundary and in close proximity to it are brighter than background.

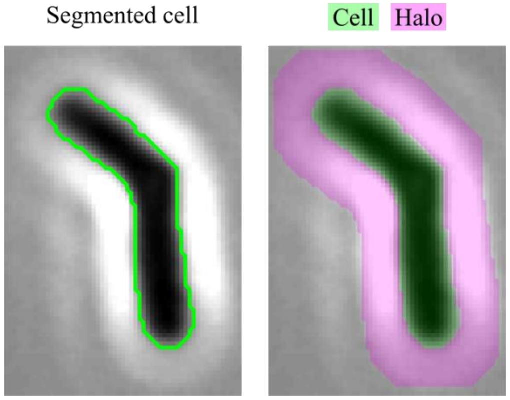

Supplementary Figure 1: Cell body and halo areas. Shown is a segmented cell with its contour represented in green and its halo region in pink.

1. Cell intensity relative to background (*CountsRelative*):

Mean brightness over all pixels within the cell boundary ($\hat{I}_{\mathrm{cell}}$) relative to the background intensity ($\hat{I}_{\mathrm{bg}}$).

$$\hat{I}_{\mathrm{cell}} = \frac{1}{N_{\mathrm{pix}}} \sum_{i=1}^{N_{\mathrm{pix}}} I_i$$

$$CountsRelative = \frac{\hat{I}_{\mathrm{cell}} - \hat{I}_{\mathrm{bg}}}{\hat{I}_{\mathrm{bg}}}$$

where, I_i is the intensity of each pixel inside the cell boundary, and N_{pix} is the total number of pixels within the cell boundary. Here, $\hat{I}_{\mathrm{bg}}$ corresponds to the mean value of all pixels in the image that are not defined as “cell” or “halo” areas (Supplementary Figure 1).

2. Cell dark pixel ratio (*NegPix*)**:**

Normalized number of pixels within the cell boundary that are darker than background (tolerance: 1 FWHM of the background intensity distribution). Numeric value between 0-1.

$$NegPix = \frac{1}{N_{\mathrm{pix}}} \sum_{i=1}^{N_{\mathrm{pix}}} I_i^-$$

$$I_i^- = \begin{cases} 1, & I_i < \hat{I}_{\mathrm{bg}} - FWHM_{\mathrm{bg}} \\ 0, & I_i \geq \hat{I}_{\mathrm{bg}} - FWHM_{\mathrm{bg}} \end{cases}$$

3. Cell bright pixel ratio (*PosPix*)**:**

Normalized number of pixels within the cell boundary that are brighter than background (tolerance: 1 FWHM of the background intensity distribution). Numeric value between 0-1.

$$PosPix = \frac{1}{N_{\mathrm{pix}}} \sum_{i=1}^{N_{\mathrm{pix}}} I_i^+$$

$$I_i^+ = \begin{cases} 1, & I_i > \hat{I}_{\mathrm{bg}} + FWHM_{\mathrm{bg}} \\ 0, & I_i \leq \hat{I}_{\mathrm{bg}} + FWHM_{\mathrm{bg}} \end{cases}$$

4. Cell mean intensity (*CellMeanInt*)**:**

Mean intensity over all pixels within the cell boundary relative to the mean background intensity.

$$CellMeanInt = \frac{\hat{I}_{\text{cell}}}{\hat{I}_{\text{bg}}}$$

5. Mean intensity of dark features (*DarkTest*):

Mean intensity over all pixels within the cell area that are darker than background relative to the mean background intensity.

$$DarkTest = \frac{1}{\hat{I}_{\text{bg}} * N_{\text{dark}}} \sum_{i=1}^{N_{\text{pix}}} I_i * I_i^{\text{D}}$$

$$I_{\text{i}}^{\text{D}} = \begin{cases} 1, & I_i < \hat{I}_{\text{bg}} \\ 0, & I_i \geq \hat{I}_{\text{bg}} \end{cases}$$

$$N_{\text{dark}} = \sum_{i=1}^{N_{\text{pix}}} I_i^{\text{D}}$$

6. **Mean intensity of bright features** (*CellBrightVal*):

Mean intensity over all pixels within the cell area that are brighter than background relative to the mean background intensity.

$$CellBrightVal = \frac{1}{\hat{I}_{\mathrm{bg}} * N_{\mathrm{bright}}} \sum_{i=1}^{N_{\mathrm{pix}}} I_i * I_i^{\mathrm{B}}$$

$$I_{\mathrm{i}}^{\mathrm{B}} = \begin{cases} 1, & I_i > \hat{I}_{\mathrm{bg}} \\ 0, & I_i \leq \hat{I}_{\mathrm{bg}} \end{cases}$$

$$N_{\mathrm{bright}} = \sum_{i=1}^{N_{\mathrm{pix}}} I_i^{\mathrm{B}}$$

7. **Under Pressure** (*UnderPressure*):

Boolean index equal to 1 if the mean intensity of bright features is larger than 1.2. This threshold value was set experimentally. This descriptor is named *Under Pressure* as bright features usually appear inside the body of *E. Coli* cells due to their turgor pressure.

8. **Halo dark pixel ratio** (*HaloNegPix*)**:**

Normalized number of pixels within the halo area (magenta in Supplementary Figure 1) that are darker than background (tolerance: 1 FWHM of the background intensity distribution). Numeric value between 0-1. Equations are equivalent to those in (2).

9. **Halo bright pixel ratio** (*HaloPosPix*)**:**

Normalized number of pixels within the halo area (magenta in Supplementary Figure 1) that are brighter than background (tolerance: 1 FWHM of the background intensity distribution). Numeric value between 0-1. Equations are equivalent to those in (3).

10. Halo mean intensity (*HaloMeanInt*)**:**

Mean intensity over all pixels within the halo area (magenta in Supplementary Figure 1) relative to the mean background intensity. Equations are equivalent to those in (4).

11. Mean intensity of dark features in halo (*HaloDarkVal*):

Mean intensity over all pixels within the halo area (magenta in Supplementary Figure 1) that are darker than background, relative to the mean background intensity. Equations are equivalent to those in (5).

12. Mean intensity of bright features in halo (*HaloBrightVal*):

Mean intensity over all pixels within the halo area (magenta in Supplementary Figure 1) that are brighter than background, relative to the mean background intensity. Equations are equivalent to those in (6).

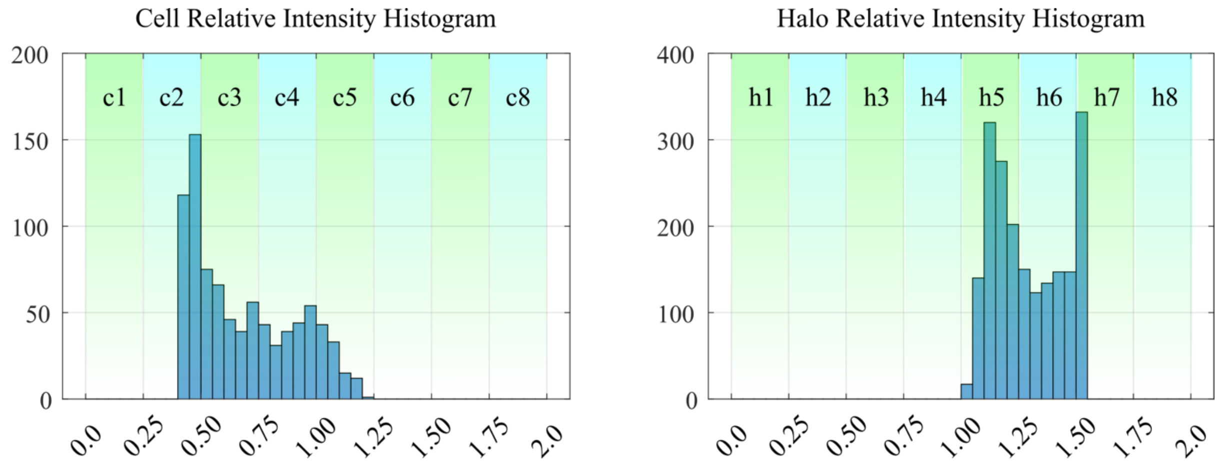

Supplementary Figure 2: Relative intensity histograms for the cell body and halo areas displayed in Supplementary Figure 1. The intensity of all pixels within the cell body and halo areas is normalized with respect to the mean background intensity.

13. Cell counts 1-8:

Array of 8 numbers summarizing the features of the intensity distribution within the cell boundary (see Supplementary Figure 2 – left). The cell count array contains the fraction of pixels whose intensity values are located between each pair of consecutive bin-edges (0.00, 0.25, 0.50, 0.75, 1.00, 1.25, 1.50, 1.75 and 2.00). Note that the upper intensity limit of 2.00 was found experimentally by examining the brightest pixels observed in wild-type experiments.

14. Halo counts 1-8:

Array of 8 numbers summarizing the features of the intensity distribution within the halo area (Supplementary Figure 2 – right). The cell count array contains the fraction of pixels whose intensity values are located between each pair of consecutive bin-edges (0.00, 0.25, 0.50, 0.75, 1.00, 1.25, 1.50, 1.75, 2.00).

Shape-based descriptors (total number: 32):

1. **Cell width (***CellWidth***):**

Width of the segmented cell calculated by the skeleton method.

2. **Perimeter length** (*Perimeter*):

Perimeter (arc length) of the segmented cell calculated by linear interpolation of the contour points.

3. **Roundness perimeter** (*Roundness_Perim*):

Perimeter of the segmented cell relative to the perimeter of a hypothetical circle featuring the same area.

4. **Roundness area** (*Roundness_Area*):

Area of the segmented cell relative to the area of a hypothetical circle featuring the same perimeter.

5. **Aspect ratio** (*Aspect_Ratio*):

Ratio between the width and the height of the segmented cell contour. The aspect ratio is estimated by first computing the minimum bounding box of the segmented contour.

6. **Max Feret** (*Max_Feret*):

Length of the largest axis of the minimum bounding box. See (5).

7. **Min Feret** (*Min_Feret*):

Length of the smallest axis of the minimum bounding box. See (5).

8. **Rectangularity** (*Rectangularity*):

Area of the segmented cell relative to the area of the minimum bouding box.

9. **Convexity** (*Convexity*):

Ratio between the perimeter of the segmented contour and its corresponding convex hull (defined as the smallest set of points on the contour that delimit a region containing all the contour points).

10. Solidity (*Solidity*):

Ratio between the area of the segmented cell relative to the area of its corresponding convex hull. See (9).

11. Elastic energy (*Elastic_Energy*):

The elastic energy of the segmented cell is estimated as follows: (i) the first derivative over the two spatial dimensions, *x* and *y*, of the idealized continuum function describing the cell contour is calculated, (ii) the resulting values are squared, and (iii) their sum is computed. Prior to these operations, cell contours are subjected to resampling for guaranteeing they are all defined by the same number of points.

12. Bending energy (*Bending_Energy*):

The bending energy of the segmented cell is estimated as follows: (i) the second derivative over the two spatial dimensions, *x* and *y*, of the idealized continuum function describing the cell contour is calculated, (ii) the resulting values are squared, and (iii) their sum is computed.

13. Fourier descriptors (*FD_1-20*):

Shape descriptors are values that convey information about the shape of an object while being invariant to as many transformations as possible, like rotations, translations, scaling, etc. On the other hand, Fourier descriptors (FD) are derived from a function called shape signature. A shape signature is a periodic 1-dimensional function that represents the boundary of a 2-dimensional area. Different shape signatures have been used to calculate FD but in general they all describe a certain property of the contour points as a function of the perimeter length. Once a shape signature has been estimated, its discrete Fourier transform is computed. In order to guarantee translation and rotation invariance, the phase information of the Fourier coefficients is ignored and only

magnitudes are kept. Scale invariance is instead attained by dividing these magnitude values by the DC component. Such normalized Fourier coefficients represent the so-called FD.

The shape signature used in this study is the centroid distance and quantifies the separation between the contour points and the centroid of the segmented shape as a function of the perimeter length. Note that the selection of the starting point along the contour does not affect the calculations due to the aforementioned translation, rotation and scaling invariance properties. For an unbiased comparison between shapes, prior to the extraction of the FD, all the segmented cell contours were smoothed and resampled in order to describe them by the same number of points. Moreover, for the purpose of comparing two different shapes we must ensure that the frequency units are the same regardless of the initial shape. To accomplish this, we describe all contours by the same number of points spaced equally along the contour curve.

In our work the contours are smoothed and resampled to contain 1000 points. Thus 500 FD can be extracted. However, we found experimentally that most shapes could be well reconstructed using about 8 FD and only the first 20 FD carried any relevant information.

Partial Least Squares Discriminant Analysis (PLSDA)

Partial Least Squares Discriminant Analysis (PLSDA) is the direct extension of Partial Least Squares regression (PLS – see forthcoming sections), developed for classification problem solving: let **X** be a $N \times J$ data matrix with J denoting e.g. the number of descriptors calculated for each of the N segmented cells. **X** is regressed via PLS on a dummy binary-coded response matrix, say **Y**, made up by a set of piled Z-dimensional row vectors (Z equals the number of categories to be discriminated), and constructed so that, if their corresponding cells are members of the z-th class, they have a 1-value in their z-th entry and 0-values in all the other ones. Whenever new cells become available, the *a posteriori* probabilities that each one of them belongs to the Z categories are calculated. The assignation is finally carried out according to either the highest-probability or the higher-than-a-threshold probability rule. The outcome of PLSDA is a linear classifier which has proved to be statistically equivalent to Linear Discriminant Analysis (LDA), but which can also be exploited when LDA cannot (e.g. when $N<J$, or when the J descriptors are strongly intercorrelated).

Soft Independent Modelling of Class Analogy (SIMCA)

Alternatively to PLSDA, Principal Component Analysis (PCA – see forthcoming sections) can be applied to every single subset of **X** (namely $\mathbf{X}_z$), including only the information associated to the cells belonging to the z-th category, so that Z independent class models are subsequently built. Unlabelled cells can be afterwards discriminated according to 2 distance indices:

- Q-statistic, which represents the perpendicular distance of each cell from the space of the z-th model;

- *D*-statistic, accounting for the distance between the projection of each cell onto the space of the *z*-th model and its origin.

Commonly, a cell is considered an outlier and thus rejected by a class model if its *reduced distance* defined as $\sqrt{\left(\frac{D}{D_{lim,Z}}\right)^2 + \left(\frac{Q}{Q_{lim,Z}}\right)^2}$ exceeds $\sqrt{2}$, where $D_{lim,Z}$ and $Q_{lim,Z}$denote an empirical confidence threshold estimated for the *z*-th category. Otherwise, the cell is recognised as being part of that particular class.

Partial Least Squares regression (PLS)

Partial Least Squares regression (PLS) is a latent variable-based statistical approach for modelling the intrinsic relationships between a matrix of predictors, say **X** ($N \times J$), and a set of response variables, **Y** ($N \times M$). The basic idea behind this technique is estimating such responses from the *A*-dimensional subspace of **X** which maximises its covariance with **Y**. Thus, the PLS structure model can be written as:

$$\mathbf{X} = \mathbf{T}\mathbf{P}^{\mathrm{T}} + \mathbf{E}$$

$$\mathbf{Y} = \mathbf{T}\mathbf{Q}^{\mathrm{T}} + \mathbf{F}$$

$$\mathbf{Y} = \mathbf{X}\mathbf{B} + \mathbf{F}$$

being **T** ($N \times A$), **P** ($J \times A$) and **E** ($N \times J$) the so-called **X**-scores, **X**-loadings and **X**-residuals matrices, respectively; **Q** ($M \times A$) and **F** ($N \times M$) the so-called **Y**-loadings and **Y**-residuals matrices, respectively; and **B** ($J \times M$) an array of regression coefficients. By PLS one does not need to assume linearly independent regressors as for most classical statistical predictive methods like Classical Least Squares (CLS).

Principal Component Analysis (PCA)

Principal Component Analysis is probably the most commonly used multivariate statistical tool to compress, describe and interpret large sets of data. Its basic principle can be summarised as follows: let **X** be a $N \times J$ matrix with J denoting the number of variables (e.g. *J sensor* responses monitored during an industrial process or J wavelengths of light scanned in a spectroscopy experiment) registered for each of N measurements performed, for instance, at N time instants or for N different individuals. When J is very large, the useful and meaningful information in **X** is usually intercorrelated among various of these variables over the whole set of recordings. Then, for a chosen degree of acceptable accuracy, it is possible to reduce the J-dimensional space of the original descriptors to an A-dimensional subspace where data mostly vary and onto which all the N objects under study can be projected and represented as new points. Mathematically speaking, PCA is based on the following bilinear structure model:

$$\mathbf{X} = \mathbf{1m}^{\mathrm{T}} + \mathbf{TP}^{\mathrm{T}} + \mathbf{E}$$

where **1** ($N \times 1$) is a vector of ones, **m** ($J \times 1$) contains the mean values of the J variables in **X**, **P** ($J \times A$) is an array of so-called *loadings*, which determine the A basis vectors (*principal components* or *factors*) of the PCA subspace, **T** ($N \times A$) defines the projection coordinates or *scores* of all the N rows of **X** on this lower-dimensional space and **E** ($N \times J$) stands for the matrix of unmodelled residuals, i.e. the portion of **X** not explained at the chosen rank, A.

The PCA solution may be formulated in many equivalent ways and attained by a variety of algorithms, among which the most widespread and popular one is certainly Singular Value Decomposition (SVD).

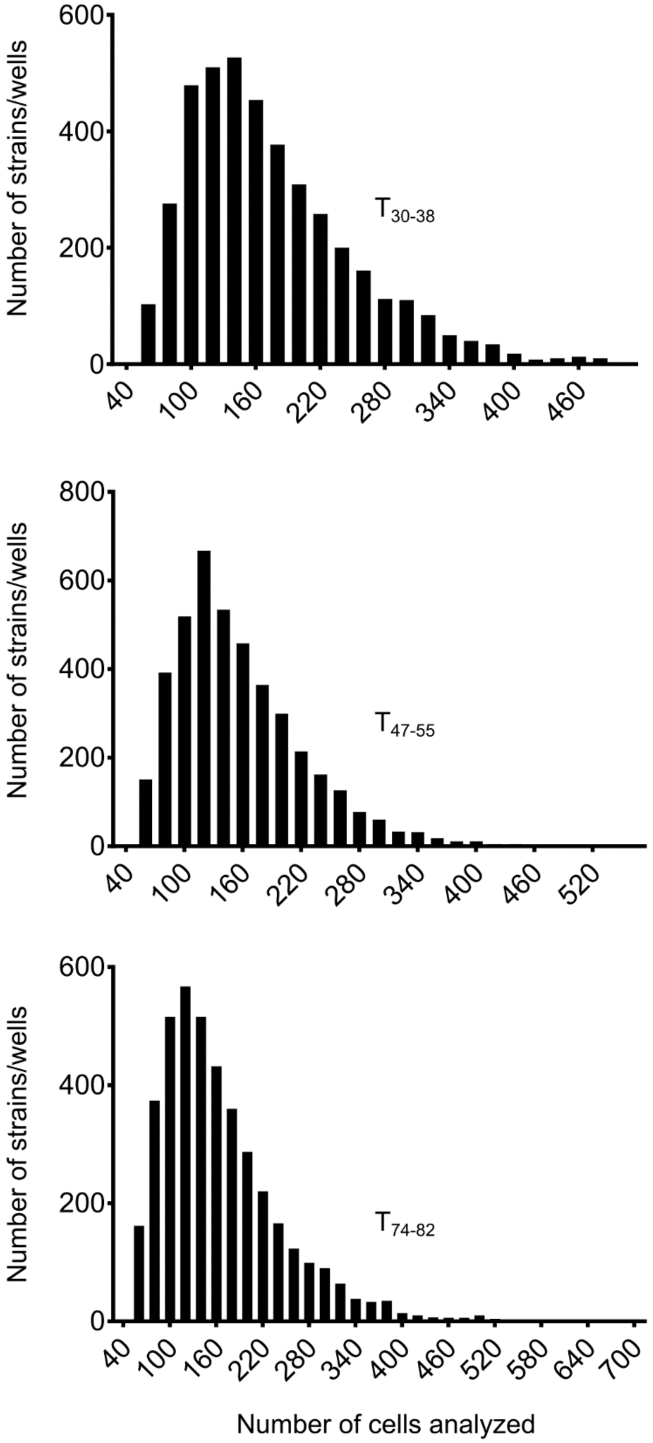

Supplementary Figure 3: Distribution of number of cells analyzed in mutants. Histograms show the number of cells analyzed for all mutants at each time-point.

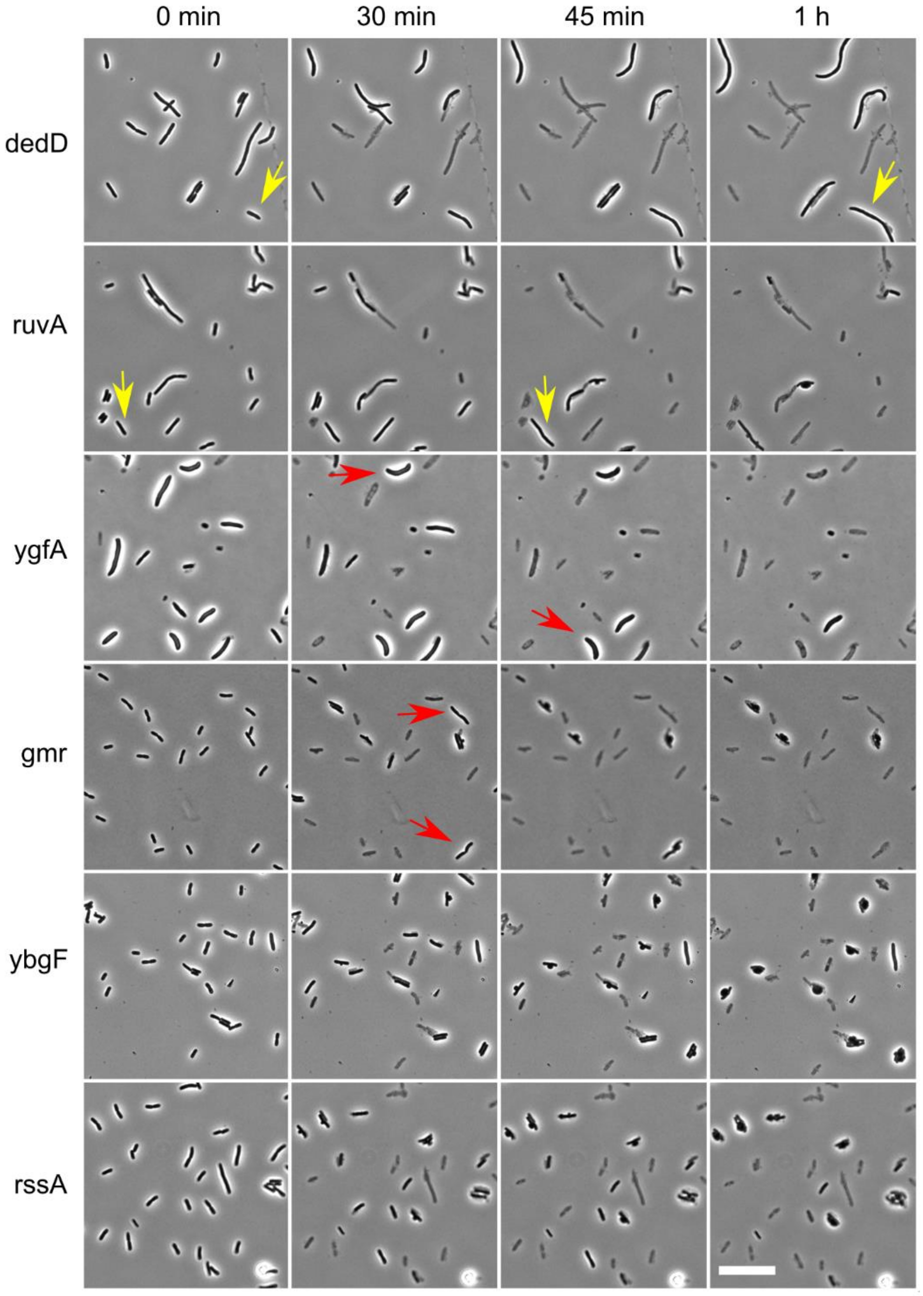

Supplementary Figure 4: Atypical morphological dynamics of mutants. Figure shows the micrographs after 0, 30, 45 and 60 min of seeding cells on cefsulodin containing agarose pads. Yellow arrows show elongation of cells and red arrows indicate cells that are curved or with constriction in the middle. Scale bar corresponds to 20 μm.

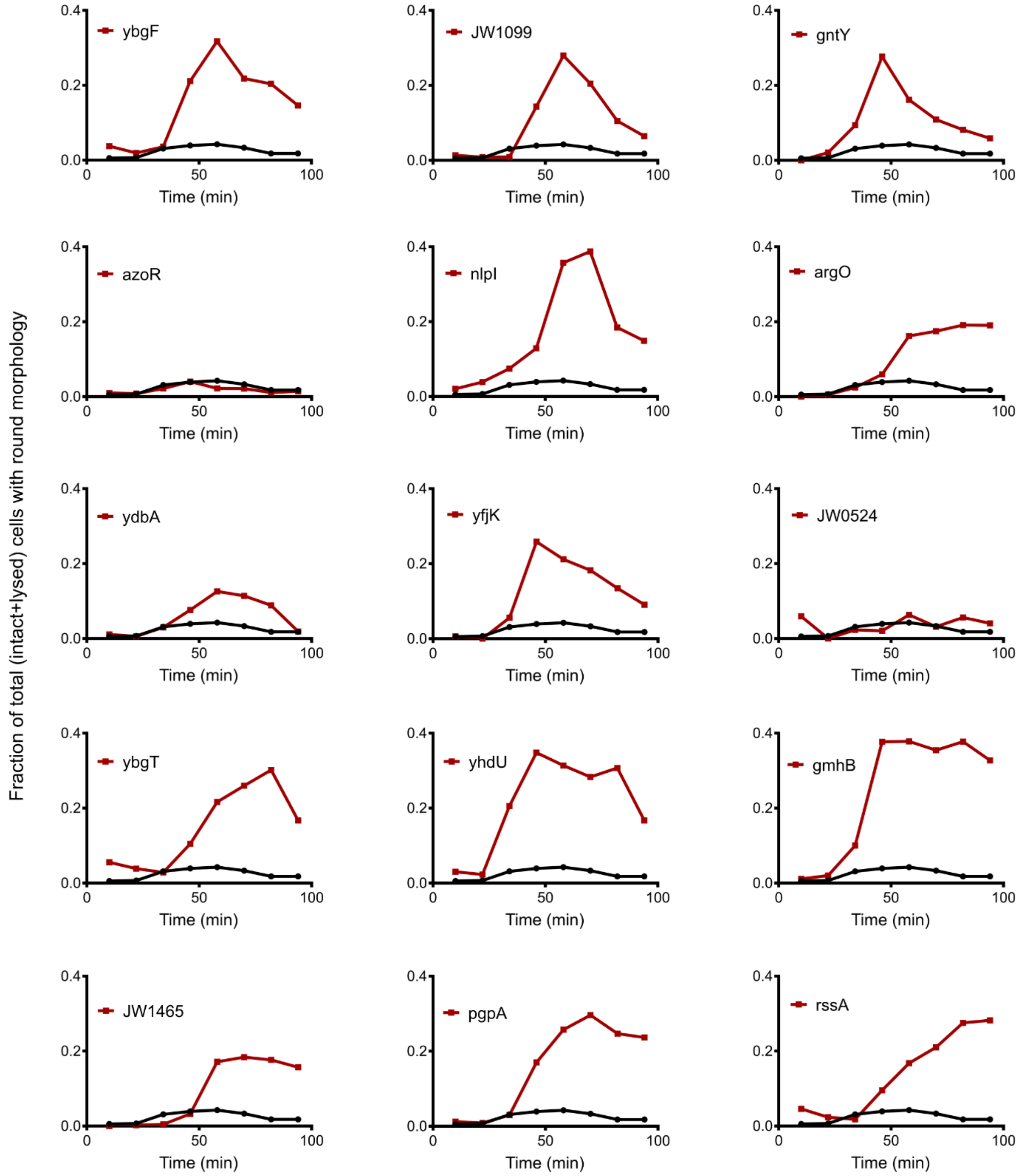

Supplementary Figure 5: Formation of cells with round morphology in stable bulging cells. Figure shows fraction of cells with round morphology in stable bulging mutants after cefsulodin treatment at t = 0. Black line represents the mean of 10 wild-type replicates and the red line represents one replicate of the corresponding mutant.

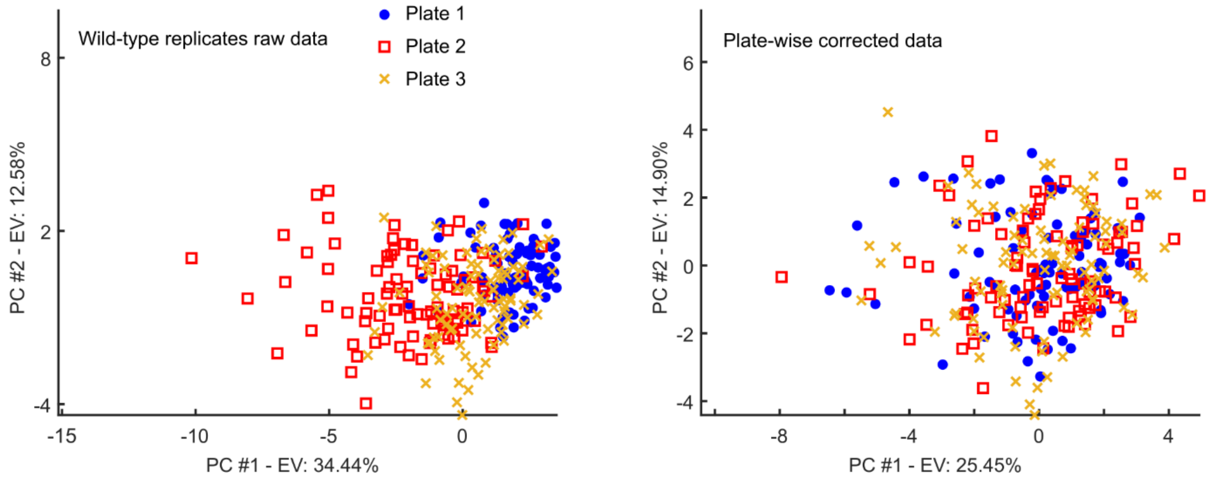

Supplementary Figure 6: Batch-effect correction. The figure shows the PCA score representation of the raw data resulting from three wild-type experiments (left) and of the same data after batch-correction (right).

Time (min)	Intact cell ratio							
10	0.881579	0.946746	0.910448	0.84472	0.939227	0.965174	0.933852	0.903846
21	0.783051	0.863248	0.747619	0.777293	0.808765	0.753731	0.774834	0.676471
32	0.275986	0.331839	0.322917	0.325359	0.316239	0.300813	0.271375	0.237838
43	0.09396	0.098039	0.146597	0.128079	0.152893	0.171315	0.097902	0.064706
54	0.092357	0.056075	0.102941	0.079646	0.083333	0.077519	0.062706	0.054545
65	0.071429	0.052133	0.04878	0.085973	0.063492	0.064639	0.043478	0.035928
76	0.034483	0.033654	0.042857	0.036697	0.050781	0.041667	0.017986	0.034682
87	0.054422	0.019048	0.029412	0.018349	0.02439	0.044177	0.024735	0.019608

Supplementary Table 1: Wild type lysis kinetics. Intact cell ratios at different time intervals from 8 experimental replicates (wells) of the wild type after treatment with cefsulodin 100 μg/ml.

Time (min)	Ratio of long cells							
10	10.000	0.197	0.171	0.178	0.215	0.197	0.131	0.167
21	21.000	0.203	0.162	0.149	0.199	0.156	0.150	0.162
32	32.000	0.214	0.209	0.100	0.325	0.300	0.313	0.233
43	43.000	0.250	0.167	0.000	0.214	0.111	0.083	0.200
Time (min)	Ratio of normal cells							
10	0.795	0.836	0.804	0.810	0.803	0.869	0.838	0.795
21	0.830	0.827	0.825	0.824	0.844	0.819	0.838	0.831
32	0.667	0.791	0.767	0.575	0.650	0.542	0.651	0.538
43	0.250	0.500	0.647	0.500	0.500	0.417	0.467	0.667
Time (min)	Ratio of small cells							
10	0.017	0.036	0.075	0.074	0.014	0.042	0.052	0.083
21	0.027	0.067	0.061	0.029	0.025	0.050	0.016	0.039
32	0.048	0.023	0.033	0.075	0.075	0.042	0.093	0.000
43	0.083	0.500	0.118	0.071	0.278	0.167	0.000	0.000
Time (min)	Ratio of round cells							
10	0.000	0.007	0.000	0.008	0.007	0.006	0.000	0.000
21	0.000	0.006	0.018	0.007	0.013	0.025	0.011	0.013
32	0.119	0.023	0.133	0.100	0.075	0.125	0.093	0.308
43	0.417	0.167	0.353	0.286	0.278	0.458	0.333	0.333

Supplementary Table 2: Dynamics of morphological changes in the wild type. Table shows the ratio of each cell morphotype at different time-points in the population of intact cells in 8 different wild type replicates after treatment with cefsulodin 100 μg/ml.

Time (h)	CFU (wild-type)			CFU (argO)		
0	4400000	6800000	5500000	5800000	5200000	3200000
0.5	1150000	1980000	870000	3200000	1800000	2200000
1	150000	220000	120000	920000	815000	1100000
2	88000	102000	84000	700000	520000	370000
Time (h)	CFU (gmhB)			CFU (rssA)		
0	7400000	5200000	5700000	7500000	3500000	4300000
0.5	4500000	2200000	3100000	5900000	1120000	3200000
1	770000	980000	570000	920000	840000	590000
2	160000	245000	320000	270000	105000	230000
Time (h)	CFU (pgpA)					
0	3200000	4400000	4800000			
0.5	430000	890000	620000			
1	100000	230000	220000			
2	62000	101000	81000			

Supplementary Table 3: Time-kill analysis of the stable bulging mutants. Table shows the colony forming units (CFU) measurements at different time intervals for the wild type and stable bulging mutants after treatment with cefsulodin 100 μg/ml.

Strains	Total cells	Recovered	Misshaped	Lysed
argO	39	35	0	4
gmhB	44	34	8	2
rssA	44	42	0	2
pgpA	30	3	11	16

Supplementary Table 4: Single-cell responses after antibiotic removal. Table shows the fates of single cells (measured by microscopy) after removal of the antibiotic for different strains. Cells that divided to at least 4 cells were classified as Recovered. Cells that could not divide and stayed abnormally shaped were classified as Misshaped.

Intact cells				Lysed cells			
Sensitivity (CV)	Specificity (CV)	Sensitivity (Test)	Specificity (Test)	Sensitivity (CV)	Specificity (CV)	Sensitivity (Test)	Specificity (Test)
97.1%	97.9%	97.9%	97.3%	97.9%	97.1%	97.3%	97.9%

Supplementary Table 5: PLSDA modelling performance. Cells were classified according to their features (or descriptors) into two classes: intact and lysed. The training set included 2017 intact cells and 1728 lysed cells.

Elongated cells				Normal cells			
Sensitivity (CV)	Specificity (CV)	Sensitivity (Test)	Specificity (Test)	Sensitivity (CV)	Specificity (CV)	Sensitivity (Test)	Specificity (Test)
90.4%	95.7%	81.4%	92.6%	90.2%	90.1%	82.5%	88.6%
Round cells				Small cells			
Sensitivity (CV)	Specificity (CV)	Sensitivity (Test)	Specificity (Test)	Sensitivity (CV)	Specificity (CV)	Sensitivity (Test)	Specificity (Test)
87.9%	96.9%	85.5%	97.6%	90.3%	98.7%	83.7%	98.5%

Supplementary Table 6: SIMCA modelling performance. Cells were classified according to their features (or descriptors) into 4 classes: elongated, normal, round and small. The training set included 376 elongated cells, 595 normal cells, 332 round cells and 258 small cells.
